# Research progress on the interaction between multiple organ-brain axes and perioperative neurocognitive disorders: a narrative review

**DOI:** 10.3389/fnagi.2025.1710009

**Published:** 2025-11-17

**Authors:** Qin Shi, Wei Wu, XiaoQin Sun, YingHai Liu, JingZheng Zeng, WeiQing Li, XueMei Dai, Gu Gong, QingQing Huang

**Affiliations:** Department of Anesthesiology, The General Hospital of Western Theater Command, Chengdu, China

**Keywords:** perioperative period, neurocognitive disorders, organ-brain axis, pathophysiological mechanism, neuroinflammation

## Abstract

Perioperative neurocognitive disorder (PND) is a common central nervous system complication during the perioperative period, characterized by memory decline, lack of concentration, and other cognitive deficits, which severely affect the quality of life and prognosis of patients. Its incidence remains high. Consequently, the prevention and treatment of PND, along with investigations into its etiology and mechanisms, have become prominent research areas. Recent studies suggest that the occurrence of PND is closely related to the interactions of multiple “important organ-brain axes,” such as the gut-brain axis, liver-brain axis, lung-brain axis, heart-brain axis, spleen-brain axis, and kidney-brain axis. Dysfunction of these axes may affect brain homeostasis through neural, immune, and endocrine pathways, leading to perioperative cognitive impairment. Although some progress has been made, the mechanisms underlying PND remain intricate and not fully elucidated. This article aims to comprehensively review how different organ systems influence central nervous system function through multifaceted interactions. It also analyzes the pathological mechanisms of PND and proposes new strategies for perioperative brain protection, with the hope of improving postoperative cognitive function and patients’ quality of life.

## 1 Introduction

Perioperative neurocognitive disorder (PND) (Fislage et al., 2024; Evered et al., 2018) refers to cognitive impairment occurring around the time of surgery, including cognitive impairment present before surgery and new cognitive decline developing within 12 months postoperatively, meeting the diagnostic criteria for neurocognitive disorders in the fifth edition of the Diagnostic and Statistical Manual of Mental Disorders (DSM-5). PND mainly includes preoperatively diagnosed neurocognitive disorders; acute postoperative delirium (POD); delayed neurocognitive recovery within 30 days postoperatively; and postoperative neurocognitive disorder (POCD) lasting up to 12 months (Evered et al., 2018). With the increasing aging population worldwide and advancements in surgical techniques, the incidence of PND and its influencing factors have become a clinical focus. Studies show that the incidence of PND varies significantly due to differences in types of surgery, patient age, and assessment methods (Connal, 2023; Ju et al., 2023; O'Gara et al., 2021; Khan et al., 2024). The incidence after non-cardiac surgery is 11–30%, while cardiac surgery [especially with cardiopulmonary bypass (CPB)] can reach 30–60% (Butz et al., 2022; Zheng et al., 2025). Advanced age (≥65 years) is a clear independent risk factor (OR 1.5–2.5), with the incidence of postoperative delirium exceeding 50% in very elderly patients (≥85 years) (Ju et al., 2023; Sun et al., 2019; Yan et al., 2019). Differences in assessment tools (neuropsychological tests, clinical diagnostic criteria, biomarker detection, etc.) lead to variability of 10–50% in reported incidence (Lewis et al., 2025; Song et al., 2023; Needham et al., 2017). Other influencing factors (Ju et al., 2023; O'Gara et al., 2021; Xu et al., 2023; Reekes et al., 2023) include pre-existing cognitive impairment, genetic factors (such as ApoEε4 genotype), pain, and anesthesia methods. Therefore, identifying high-risk patients and implementing corresponding preventive measures is of great significance for reducing the incidence of PND.

In recent years, research has gradually revealed several pathophysiological processes of PND, with many focusing on the neuroinflammatory cascade activation caused by a single ‘organ-brain axis.’ Surgical trauma releases damage-associated molecular patterns (DAMPs), which activate the innate immune system. This activation leads to significantly elevated levels of pro-inflammatory cytokines (TNF-α, IL-6), increasing by 2- to 3-fold within 24 h post-surgery (Subramaniyan and Terrando, 2019; Safavynia and Goldstein, 2019; Festa et al., 2025; Yang et al., 2024). These changes disrupt the integrity of the blood–brain barrier (BBB), activate microglia, and consequently lead to hippocampal neuronal apoptosis and cognitive dysfunction. The humoral factors or neural reflexes produced by organs such as the intestine, liver, and spleen may participate in multiple stages of this process. For example, the gut microbiota regulates neuroinflammation and cognitive function through the brain-gut axis (Que et al., 2024; Zhang X. et al., 2024); liver metabolic dysfunction can exacerbate systemic inflammation and the accumulation of neurotoxic substances through the liver-brain axis (López-Franco et al., 2021); and dysfunction of important organs such as the heart (Liu X. et al., 2024), lungs (Smith et al., 2014), and kidney (Davani et al., 2021) can also contribute to the occurrence and development of PND by affecting cerebral blood flow perfusion, oxygenation, and toxin clearance.

It is worth noting that the dysregulation of different organ systems may differentially affect the clinical trajectory of PND through spatiotemporally specific inflammatory and metabolic cascade reactions (Liu et al., 2025; Seki et al., 2021; Figueiredo and Devezas, 2024). For example, gut microbiota dysbiosis may rapidly induce acute neuroinflammation via neuroimmune pathways, which is closely related to the occurrence of POD (Garcez et al., 2023), whereas liver metabolic dysfunction may be more likely to lead to persistent cognitive decline through accumulated systemic low-grade inflammation (Ingustu et al., 2023). This sequential “synergistic attack” mechanism across multiple organ systems over time may explain why single organ-targeted interventions have limited efficacy in clinical trials. Therefore, existing research focusing on the pathological mechanisms of PND affecting brain cognition in single organ systems is insufficient to elucidate the changes in cognitive function throughout the perioperative period.

The existing literature has not sufficiently clarified how different organ-brain axes dynamically interact and form network cascades under perioperative stress, ultimately leading to PND. This multi-organ interaction network may provide a new theoretical framework for early warning and precise intervention of PND. Therefore, this review aims to go beyond a mere enumeration of single axes and systematically focus with an emphasis on the integrative mechanisms of the multi-organ-brain network in PND. It highlights the synergistic or antagonistic effects between various organ axes and how they influence clinical cognitive outcomes through common pathways such as neuroinflammation, metabolic disorders, and exosomal communication. By critically synthesizing existing fundamental and clinical research evidence, we aim to clarify current knowledge gaps regarding cognitive outcomes and provide a new conceptual basis for constructing a multi-organ risk-based PND prediction model and multi-target integrated intervention strategies.

## 2 Methods

This review aims to systematically summarize the effects and mechanisms of several key organ-brain axes—such as the gut-brain, lung-brain, and heart-brain axes—on PND by conducting a systematic literature search and screening to comprehensively gather relevant evidence.

### 2.1 Literature search strategy

The search time frame includes studies published up to August 2025, with particular emphasis on recent research progress. Specifically, the databases searched are PubMed, MEDLINE, EMBASE, Google Scholar, Cochrane Library, and China National Knowledge Infrastructure (CNKI). The search strategy comprehensively uses the following keywords and their synonyms and related terms: “perioperative neurocognitive disorders”; “PND”; “postoperative cognitive dysfunction”; “pathogenic mechanism”; “gut”; “hepatic”; “lung”; “cardiac”; “kidney”; “spleen”; and “inflammation.” Boolean operators (AND, OR) are employed for combination and refinement.

### 2.2 Inclusion and exclusion criteria for literature selection

To clarify the scope of the study, the following criteria are established:

Inclusion criteria: (1) The study subjects are patients undergoing surgery or perioperative animal models relevant to the surgical procedures studied; (2) The study focuses on the interaction between one or more important organs (intestines, liver, lungs, heart, spleen, kidney) and the brain. It also explores the mechanisms underlying their association with PND, including POD and POCD, or management strategies for PND; (3) The study type is clinical research (randomized controlled trials, cohort studies, case–control studies, etc.) or basic experimental research (animal models); (4) Full text available.

Exclusion criteria: (1) Non-Chinese or English literature; (2) Conference abstracts, reviews, case reports, and studies for which the full text cannot be obtained; (3) Studies without direct relevance to the organ-brain axis or the pathological mechanisms of PND; (4) Studies with incomplete data or insufficient information for effective analysis.

### 2.3 Literature screening and data extraction process

The literature screening was conducted independently by three authors, YHL, XQS, and XMD, to ensure the repeatability and objectivity of the process. The specific process was as follows:

(1) Initial screening: Studies that clearly did not meet the inclusion criteria were excluded based on titles and abstracts.
(2) Secondary screening: The full texts of the remaining studies were read, and strict screening was conducted based on the predetermined inclusion and exclusion criteria.
(3) Discrepancy resolution: At any stage of screening, if there was any disagreement, it was first resolved through discussion and negotiation among the three reviewers. If consensus could not be reached after discussion, the fourth author (QS) made the final decision.

After screening, the final list of included studies was jointly reviewed and approved by all authors.

### 2.4 Bias control and literature quality consideration

Although this study is a systematic review rather than a meta-analysis and did not formally score the quality of the included studies, we strive to control bias and carefully assess the strength of evidence through the following structured process.

Standardization of process: Following the principles of systematic reviews, we developed and implemented clear search strategies, inclusion and exclusion criteria and screening processes.

Assessment of evidence sources: In the analysis and discussion of results, we fully considered the inherent limitations of different types of studies. For example, we maintained necessary caution when interpreting mechanistic relationships and deriving clinical implications from evidence derived from animal experiments or small-sample clinical observational studies, especially in emerging fields such as the spleen-brain axis and kidney-brain axis. We also clearly pointed out that these findings require validation through large-scale prospective studies.

Attention to research heterogeneity: We recognize that the heterogeneity of PND diagnostic criteria, assessment tools, and follow-up times may affect the comparability between studies, and we accounted for this factor when synthesizing evidence.

### 2.5 Statistical effect size indicators

To present the research results clearly and quantitatively, this review will uniformly adopt the following statistical effect size indicators when summarizing the literature findings: Odds Ratio (OR) is used to measure the strength of association of risk factors, where OR > 1 indicates increased risk. Prevalence Ratio (PR) is used to compare the prevalence of disease between groups, with PR > 1 indicating higher disease prevalence. Standardized Mean Difference (SMD) quantifies the magnitude of difference in continuous variables (such as behavioral scores) between two groups. Pearson correlation coefficient (R) and Spearman rank correlation coefficient (RS) are used to assess the strength and direction of linear and monotonic relationships between two continuous variables, respectively. Standardized regression coefficient (β) indicates the independent effect and direction of independent variables on dependent variables in multivariate linear regression analysis. In addition, the *p*-value is used to assess whether the observed effect is statistically significant, typically with a threshold of (*p* < 0.05). The following text will quantitatively synthesize the evidence using these effect size indicators to enhance the objectivity and comparability of the conclusions.

**Figure 1:**
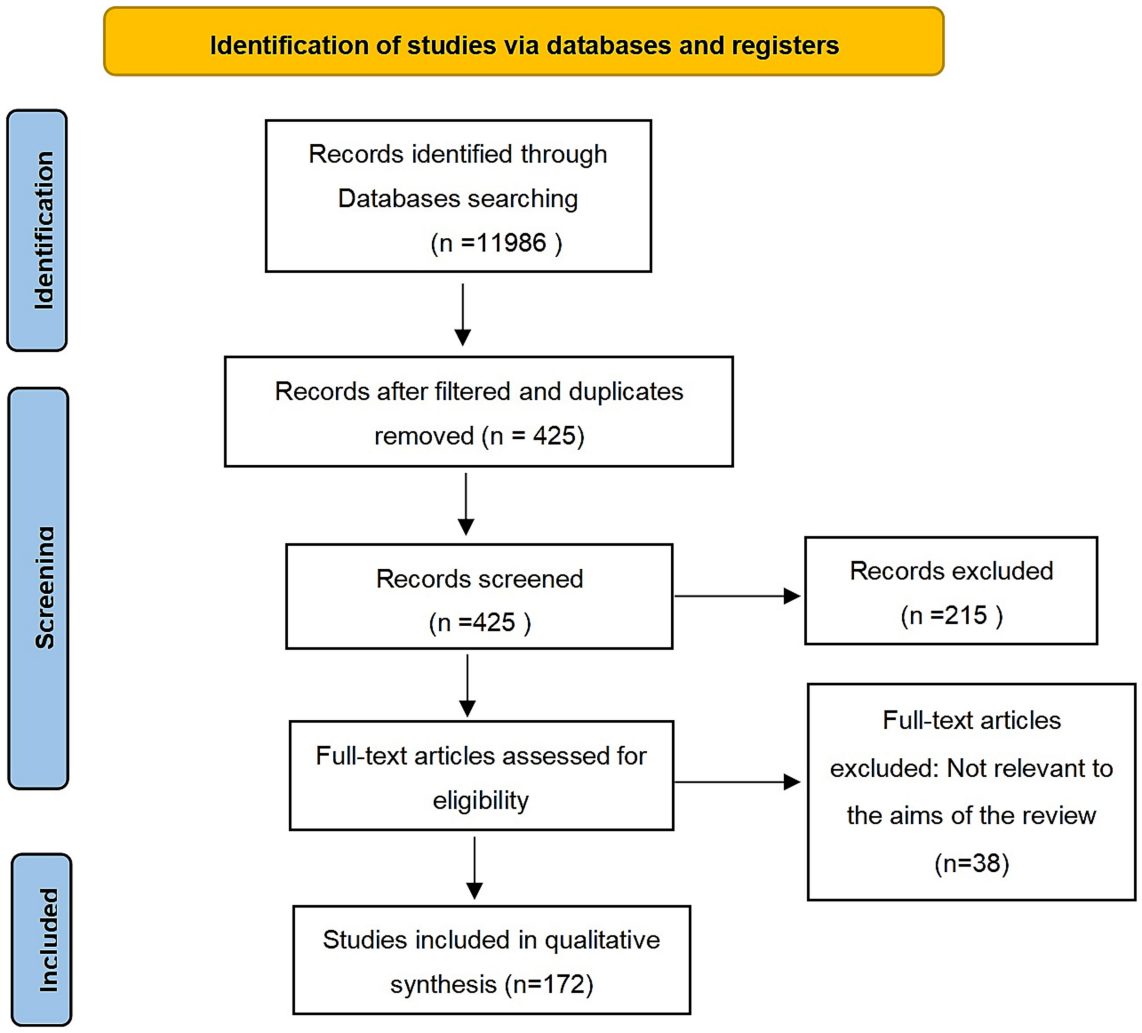
PRISMA-2020 flow diagram for selection of studies. Source: Page et al. (2021). This work is licensed under CC BY 4.0. To view a copy of this license, visit https://creativecommons.org/licenses/by/4.0/.

The final search yielded a total of 1,986 articles. After filtering and removing duplicates, 425 articles remained. We assessed their eligibility and excluded 215 articles. From the remaining 210 full-text articles, 172 were included based on predefined criteria of novelty and relevance (Figure 1).

## 3 Multiple organ-brain axes and their relationship with PND

### 3.1 The role of the gut-brain axis in PND

The gut microbiota (Gomaa, 2020) refers to the complex microbial community that colonizes the human digestive tract, mainly composed of bacteria, fungi, viruses, and archaea. These microorganisms form a symbiotic relationship with the host and participate in various physiological functions such as digestion, metabolism, immune regulation, and neural signal transmission (Gomaa, 2020). There is a bidirectional communication network between the gut microbiota and the central nervous system (CNS), involving neural, endocrine, and immune pathways, all of which play important regulatory roles in the gut-brain axis. Neural pathways include the transmission of microbial metabolites to the CNS via the vagus nerve (Dalile et al., 2019). Endocrine pathways involve the gut microbiota influencing the synthesis of 5-hydroxytryptamine (5-HT) and gamma-aminobutyric acid (GABA), thereby regulating neurotransmitter balance (Qu et al., 2024). Immune pathways (Dalile et al., 2019; Rutsch et al., 2020) are characterized by dysbiosis leading to the translocation of bacterial toxins into the bloodstream, which triggers systemic and neural inflammation.

#### 3.1.1 Gut microbiota—gut-brain axis

Multiple factors (Mc Loughlin and Hinchion, 2023) during the perioperative period can significantly affect the diversity, function, and homeostasis of the gut microbiota. First, the type and duration of surgery play important roles. Compared to minor and shorter surgeries, major surgeries such as cardiac and abdominal procedures have a higher probability of causing dysbiosis. Additionally, the longer the duration of CPB, the higher the level of plasma lipopolysaccharide (LPS), which exacerbates systemic inflammation. Stein-Thoeringer CK (Stein-Thoeringer et al., 2025) and colleagues found that gastrointestinal surgeries, such as pancreatoduodenectomy, can lead to excessive proliferation of enterococci, thereby increasing the risk of postoperative infections. Furthermore, anesthesia and medications also influence the gut microbiota. General anesthetics, such as sevoflurane (Liu et al., 2022), can increase the likelihood of dysbiosis, whereas regional anesthesia, such as epidural anesthesia, has a less disruptive effect on the microbiota and may be more protective. The use of antibiotics, such as cephalosporins (Yin et al., 2025), during the perioperative period can significantly reduce the diversity of gut microbiota and promote the colonization of drug-resistant bacteria. Luo et al. (2021) found in studies on elderly mice that cefazolin sodium can alter the gut microbiome and increase the permeability of the BBB, thereby affecting postoperative cognitive function. Patient factors, including age and nutritional status, also influence the homeostasis of the gut microbiota. Research by Liufu et al. (2020) indicates that the alterations in gut microbiota caused by surgical anesthesia exhibit age-dependent changes, characterized by a significant decrease in both the abundance and diversity of the microbiota with increasing age. All of the aforementioned factors can contribute to a decline in cognitive function by affecting the composition and function of the gut microbiota.

#### 3.1.2 The role of the gut-brain axis dysfunction in the pathogenesis of PND

Neuroinflammation is considered a major hallmark of PND (Subramaniyan and Terrando, 2019; Yang et al., 2024). The pathophysiological changes during the perioperative period and the stress response induced by surgical anesthesia may lead to dysbiosis of the gut microbiota, which in turn causes central inflammation through neural, immune, and endocrine pathways, ultimately resulting in the occurrence of PND. (1) Immune pathways. Perioperative stress, anesthesia, and antibiotic use can lead to dysbiosis of the gut microbiota, characterized by an increased ratio of enterococci to bacilli and elevated plasma LPS levels. A prospective study showed that intravenous injection of LPS is sufficient to directly induce significant systemic inflammatory responses in healthy volunteers, subsequently leading to decreased attention, memory, and a delirious state (van den Boogaard et al., 2010). This demonstrates that the translocation of gut-derived LPS is one of the key initiating factors of perioperative neuroinflammation and delirium. Mechanistically, LPS and the subsequent increase in pro-inflammatory factors can disrupt the integrity of the BBB. A critical experimental study showed that lateral ventricle injection of LPS significantly reduced the expression level of the tight junction core protein claudin-5 in the microvessels of the mouse cortex by about 40% within 24 h (Banks et al., 2015). It also increased the permeability of the blood–brain barrier to sucrose by approximately 75% (Banks et al., 2015). Interventional studies have confirmed that supplementation with probiotics such as Lactobacillus and Bifidobacterium not only restores gut homeostasis but also reduces the levels of inflammatory factors in the hippocampus by about 35–45%, significantly improving performance in cognitive behavioral tests—exploratory behavior in the elevated plus maze and open field test is enhanced by approximately 50–60% (Pei et al., 2024). (2) In terms of neural pathways, the metabolic products of gut microbiota, such as short-chain fatty acids (SCFAs), and the vagus nerve are key mediators. Brain-derived neurotrophic factor (BDNF) (Albini et al., 2023) maintains the growth and development of brain neurons. However, under stress conditions, perioperative dysbiosis of gut microbiota can be transmitted to the brain via the vagus nerve, inhibiting the expression of BDNF in hippocampal neurons. This makes neurons more susceptible to oxidative stress damage, thereby leading to changes in cognitive function. At the same time, gut microbial metabolites (Dalile et al., 2019), such as SCFAs, directly activate vagal afferent fibers, transmitting signals to the nucleus of the solitary tract. This, in turn, affects emotion- and cognition-related brain regions such as the amygdala and hippocampus. SCFAs (Que et al., 2024; Wu et al., 2020) improve cognitive function by inhibiting neuroinflammation and promoting neurogenesis. Animal experiments (Bravo et al., 2011) have shown that vagotomy can completely block the anxiolytic effects produced by probiotics (such as *Lactobacillus rhamnosus*), demonstrating the necessity of this pathway in gut-brain communication. (3) Endocrine pathways. Certain intestinal epithelial cells have endocrine or paracrine functions, such as enterochromaffin cells that produce 5-hydroxytryptamine, a precursor of the central neurotransmitter 5-HT (Shin and Kim, 2023). Surgical anesthesia disrupts gut microbiota and impairs the synthesis and metabolic homeostasis of neurotransmitters involving intestinal endocrine cells and gut microbiota (Qu et al., 2024). This disruption can lead to fluctuations in 5-HT and GABA levels, thereby affecting perioperative mood, behavior, and learning and memory abilities. The gut microbiota can also regulate the secretion of glucagon-like peptide-1 (GLP-1) and peptide YY (PYY), and receptors for these peptides are widely distributed in the hippocampus and prefrontal cortex, affecting appetite and cognitive function (Tolhurst et al., 2012). GLP-1 enhances synaptic plasticity through the cAMP-PKA pathway. Perioperative gut dysfunction can lead to a decrease in postoperative GLP-1 levels, which is significantly related to memory impairment (Ren et al., 2024).

**Figure 2:**
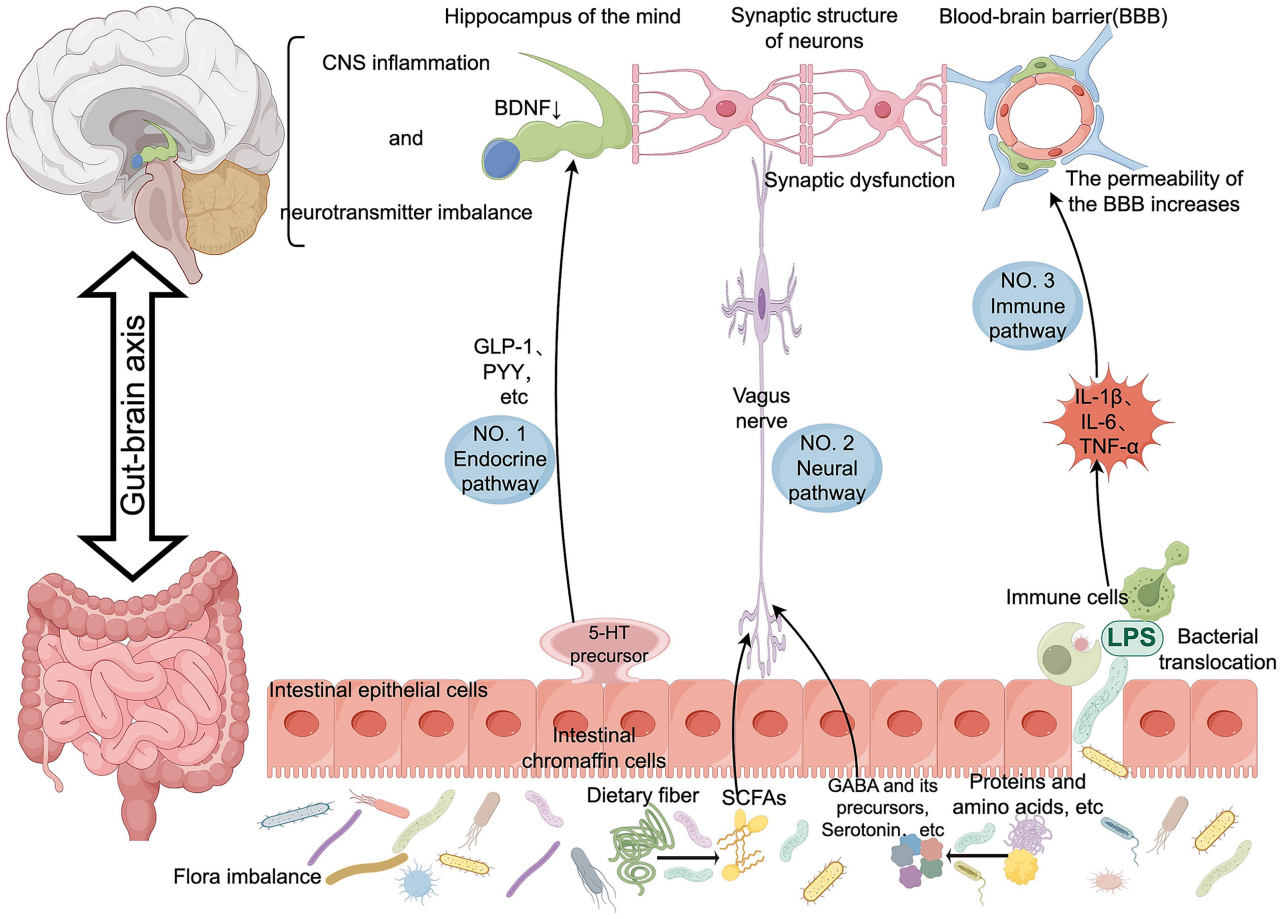
The role of the gut-brain axis in PND. Authors declare no competing interests. Graphical figures were created with FigDraw software (https://www.figdraw.com/, accessed on August 31, 2025). Copyright Code: RORSR00efe.

In summary, physiological changes during the perioperative period and stress responses triggered by surgical procedures may lead to dysbiosis of the gut microbiota, thereby affecting brain function (Figure 2). Therefore, regulating gut microbiota may help alleviate perioperative neurocognitive disorders and improve patients’ cognitive function and quality of life.

### 3.2 The role of the liver-brain axis in PND

The liver is not only an important organ for metabolism and detoxification, but recent studies have shown that liver dysfunction may exacerbate the development of PND through mechanisms involving the liver-brain axis. These mechanisms may include metabolic imbalance, systemic inflammation, and disruption of the gut-liver-brain axis. Such disturbances can worsen postoperative neurocognitive dysfunction (Park et al., 2017).

#### 3.2.1 Imbalance of liver metabolites and neurotoxicity

Recent studies have shown that the types, concentrations, and dynamic balance of liver metabolites are closely related to neurocognitive function. The disruption of key metabolites such as SCFAs, bile acids, ammonia, and ketone bodies has been confirmed to have a significant association with the occurrence and development of cognitive dysfunction, neurodegenerative diseases, and mental disorders.

SCFAs, primarily produced by gut microbiota fermentation of dietary fibers (such as acetate, propionate, and butyrate), can cross the blood–brain barrier and directly act on microglia and neurons. They regulate the activity of histone deacetylase enzymes (HDACs) and promote the expression of BDNF in the brain, thereby improving synaptic plasticity as well as learning and memory functions (Silva et al., 2020). In chronic liver diseases such as cirrhosis, dysbiosis of the gut microbiota often leads to a significant reduction in SCFA levels. SCFAs are neuroprotective, and their deficiency is considered one of the important factors contributing to related cognitive impairment. Therefore, supplementation with butyrate is regarded as a promising therapeutic strategy (Dalile et al., 2019). Animal experiments have shown that a reduction in SCFAs can damage the blood–brain barrier and promote glial cell activation; however, supplementation with exogenous butyrate can significantly alleviate blood–brain barrier damage in model mice (Xiao et al., 2022). Furthermore, clinical studies have indicated that in patients with cirrhosis, circulating butyrate levels have a moderate to strong significant negative correlation with key inflammatory mediators, LPS and IL-6 (LPS: *RS* = −0.58; IL-6: *RS* = −0.49; both *p* < 0.001) (Juanola et al., 2019). Thus, the deficiency of butyrate associated with liver disease constitutes an important upstream risk factor for perioperative cognitive impairment. In addition, SCFAs may influence mood and decision-making functions by activating free fatty acid receptors (FFAR2/3) to regulate the activity of serotonergic neurons (Yano et al., 2015).

Bile acids are the end products of cholesterol metabolism in the liver. Recent studies have found that they can regulate neuroinflammation and mitochondrial function by activating the Farnesoid X receptor (FXR) and the TGR5 receptor (Xie et al., 2018). When liver function is impaired, the accumulation of secondary bile acids, such as deoxycholic acid, can induce excessive activation of microglia. This activation promotes the release of IL-1β through the NF-κB pathway, thereby exacerbating neuroinflammation (MahmoudianDehkordi et al., 2019). In contrast, ursodeoxycholic acid (UDCA) has neuroprotective effects by inhibiting α-synuclein aggregation. Moreover, clinical trials have suggested that UDCA can slow cognitive decline in early Parkinson’s disease (PD) patients (Qi et al., 2021).

Ammonia is a product of protein metabolism that involves the liver. When liver function is impaired, accumulated ammonia can interfere with the activity of glutamine synthetase in astrocytes, disrupt neurotransmitter cycling, induce brain edema and bioenergetic dysfunction, directly damage the hippocampus and prefrontal cortex, and affect memory and executive function (Hadjihambi et al., 2017). In addition, hyperammonemia may also lead to calcium overload by activating NMDA receptors, promote pathological tau protein hyperphosphorylation, and contribute to Alzheimer’s disease-like changes (Rodrigo et al., 2010), ultimately resulting in neurocognitive dysfunction. Although hyperammonemia is considered one of the core mechanisms underlying delirium in patients with liver disease, its general applicability as a predictive indicator should be viewed with caution. A systematic review and meta-analysis focused on liver transplant patients clearly pointed out that preoperative ammonia levels have no significant correlation with the occurrence of postoperative delirium. In contrast, the patient’s baseline liver function status, indicated by high Child-Pugh or MELD scores, advanced age, history of preoperative hepatic encephalopathy, and duration of surgery are more definitive risk factors (Zhou et al., 2021).

In addition, abnormal liver function causes metabolic disorders involving metal ions such as manganese and copper, leading to excessive generation of free radicals, inhibiting the activity of superoxide dismutase (SOD) and inducing lipid peroxidation in neurons (Jiang et al., 2009), directly affecting the function of the CNS.

#### 3.2.2 Systemic inflammation and neuroinflammation induced by liver dysfunction

Liver dysfunction can amplify systemic inflammatory responses. Patients with liver dysfunction during the perioperative period exhibit higher levels of inflammation compared to healthy individuals, accompanied by intestinal barrier damage and gut microbiota dysbiosis. LPS entering the liver activates Kupffer cells, which then release increased amounts of pro-inflammatory cytokines (Yang et al., 2024; Liu et al., 2022; Fukui, 2015). These pro-inflammatory cytokines signal through the vagus nerve or directly cross the BBB, causing central inflammation and impairing synaptic plasticity (Llansola et al., 2024). Animal experiments have confirmed that mice with chronic liver conditions, such as cirrhosis, show significantly elevated levels of IL-1β in the hippocampus after surgical trauma, which is associated with spatial memory impairment (Cao et al., 2010). Clinical data indicate that liver disease patients with elevated preoperative IL-6 levels have more than double the risk of developing postoperative delirium (*OR* > 2.0) (Capri et al., 2014). Based on this mechanism, preoperative anti-inflammatory interventions in liver disease patients have shown promising potential. However, a randomized controlled trial in liver resection patients demonstrated that although preoperative administration of high-dose corticosteroids significantly suppressed the intraoperative increase of IL-6 levels by over 50%, it did not significantly reduce the overall incidence of postoperative delirium (Awada et al., 2022). This suggests that the pathophysiology of delirium during liver resection may involve more complex inflammatory mechanisms beyond IL-6 that warrant further investigation.

#### 3.2.3 Gut-liver-brain axis dysfunction

In section 3.2.2, it is mentioned that SCFAs derived from gut microbiota, such as butyrate (Silva et al., 2020; Dalile et al., 2019), are absorbed into the liver via the portal vein and then distributed to the brain through hepatic blood circulation. These SCFAs exhibit neuroprotective effects by inhibiting HDAC activity and promoting BDNF expression. Dysbiosis of gut microbiota leads to a metabolic imbalance and reduced levels of SCFAs in patients with liver disease, which may weaken their inhibitory effects on neuroinflammation (Xu et al., 2024). The role of the gut-liver-brain axis—comprising gut barrier disruption, endotoxin translocation, impaired hepatic detoxification, and the resulting neuroinflammation cascade—in central inflammation and neurocognition is becoming clearer. Recent clinical trials have shown that perioperative supplementation with probiotics can reduce the incidence of delirium by 48%, suggesting that regulating the gut-liver-brain axis is a potential intervention strategy (Fukui, 2015).

#### 3.2.4 Liver and anesthesia

The risk of cognitive changes significantly increases in patients with hepatic dysfunction after receiving anesthesia. In hepatic dysfunction, the sensitivity of GABA receptors is enhanced, while the metabolism of dopamine and serotonin is abnormal, which may lead to a postoperative imbalance between excitation and inhibition (Hadjihambi et al., 2017). The metabolism of anesthetic drugs, such as benzodiazepines and propofol, depends on hepatic enzymes, such as CYP450. In patients with impaired liver function, the clearance rate decreases, prolonging the duration of drug action and further exacerbating cognitive suppression (Elvir-Lazo et al., 2021; Tsai et al., 2015). These findings demonstrate that liver function affects cognitive function in patients after anesthesia. Retrospective analyses also show that the incidence of cognitive impairment after surgery is significantly higher in patients with Child-Pugh grade B or C cirrhosis compared to those with grade A, and this is significantly related to the dosage of anesthetic drugs used during surgery (Zhou et al., 2023).

**Figure 3:**
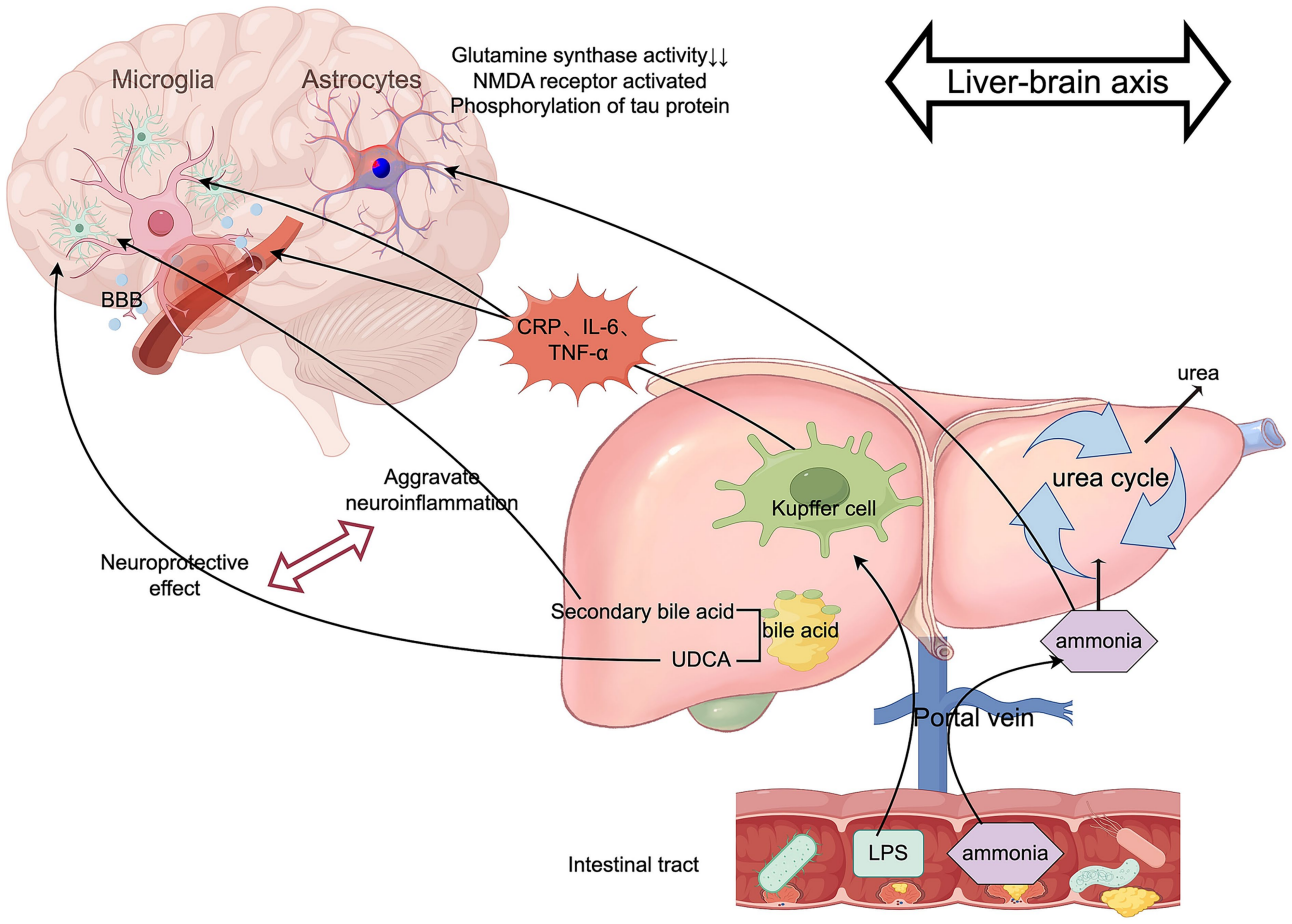
The role of the liver-brain axis in PND. Authors declare no competing interests. Graphical figures were created with FigDraw software (https://www.figdraw.com/, accessed on August 31, 2025). Copyright Code: TWAUA9aba9.

In summary, hepatic dysfunction affects the CNS through metabolic, inflammatory, and pharmacokinetic pathways, significantly increasing the risk of PND (Figure 3).

### 3.3 The role of the lung-brain axis in PND

The decline in pulmonary function is closely associated with cognitive dysfunction, especially in the elderly population. Studies show that patients with chronic obstructive pulmonary disease (COPD) often experience cognitive decline. The prevalence of mild cognitive impairment (MCI) in these patients reaches 32.1%, which is significantly higher than the 14.5% observed in matched control populations (*PR* = 2.2) (Zhang et al., 2025). Furthermore, respiratory function is consistently negatively correlated with overall cognitive function; for example, the correlation coefficient between percent predicted FEV_1_ and MoCA score is *R* = 0.28 (*p* < 0.01) (Tachibana et al., 2024). This association is particularly significant in specific cognitive domains such as attention and memory, with a larger effect size observed for memory impairment (β = 0.35, *p* < 0.001) (Annaka et al., 2022). Moreover, the lung-brain axis may be involved in the occurrence and development of PND, through pathways such as disorders of brain energy metabolism, changes in neural excitability, and inflammatory neural damage.

#### 3.3.1 The impact of hypoxemia on brain energy metabolism disorders

The brain tissue is the organ with the highest oxygen consumption in the human body (accounting for 20% of total oxygen consumption), and its energy supply almost entirely relies on aerobic metabolism (Amin et al., 2024; Li C. et al., 2022). Surgery-related respiratory suppression, the effects of anesthetic drugs, or pre-existing pulmonary diseases can lead to perioperative hypoxia in patients. When the arterial partial pressure of oxygen (PaO_2_) falls below 60 mmHg or chronic hypoxia (SpO_2_ < 90% sustained for more than 5 min) occurs (Ding et al., 2018; Zhang K. et al., 2024; Jing et al., 2022), mitochondrial function in the hippocampus is impaired, resulting in a 40–60% reduction in ATP production. Insufficient ATP leads to the inhibition of presynaptic glutamate transporter function, causing extracellular glutamate concentration to increase several dozen-fold (Jing et al., 2022), which in turn triggers the degradation of neuronal cytoskeletal proteins. At the same time, during hypoxia, brain tissue initiates glycolysis, and lactic acid production increases by 3–5 times. This metabolic acidosis further inhibits GABA aminotransferase activity, affecting the metabolism of inhibitory neurotransmitters (Mácha et al., 2024). Clinical studies have found that serum levels of hypoxia-inducible factor HIF-1α are significantly higher in patients with obstructive sleep apnea (OSA) who develop postoperative delirium (*p* < 0.01); elevated HIF-1α is an independent risk factor for PD (*OR* = 4.82) (Breitkopf et al., 2025). Additionally, the duration of mild hypoxemia (55 mmHg ≤ PaO_2_ < 60 mmHg) accumulated during surgery is significantly associated with an increased risk of postoperative delirium. For every additional 10 min of cumulative exposure time, the risk of developing postoperative delirium increases by approximately 18% (*OR* = 1.18) (Ahrens et al., 2023). These findings indicate that preoperative chronic hypoxia load (reflected by hypoxia biomarkers) lays a pathological basis for the occurrence of delirium. Moreover, intraoperative hypoxia greatly enhances the brain’s vulnerability to perioperative stress. Together, these factors lead to the occurrence of PND. Therefore, does monitoring regional cerebral oxygen saturation (rSO_2_) during the perioperative period have value in predicting and preventing postoperative delirium? Some studies suggest that a decrease in brain oxygen is a risk factor; however, there are also high-quality prospective observational studies that have yielded negative results, finding no association between perioperative rSO_2_ parameters and delirium risk (Fischer-Kumbruch et al., 2022). This inconsistency may be related to the heterogeneity of the study population, types of surgery, and methods of delirium assessment.

#### 3.3.2 Blood carbon dioxide levels and their effects on neural excitability

Perioperative respiratory dysfunction leads to abnormal levels of carbon dioxide in the blood, either hypercapnia or hypocapnia, each of which is considered an important modifiable factor that induces and promotes PND. The core mechanism lies in the fact that carbon dioxide (Meng and Gelb, 2015), a potent vasodilator and neuromodulator that can freely penetrate the BBB, significantly affects neuronal excitability through direct and indirect pathways. This interference disrupts the normal function of cognitive-related brain regions.

Hypocapnia has been supported by multi-level evidence as a clear risk factor for PND. Early studies found that intraoperative end-tidal hypocapnia (EtCO_2_ < 30 mmHg) is an independent predictor of postoperative delirium (*OR* = 3.2, 95% *CI* 1.2–8.9) (Mutch et al., 2018). This finding was subsequently confirmed in more precise studies: the latest evidence shows a clear dose-dependent relationship between intraoperative hypocapnia (PaCO_2_ < 30 mmHg) and the risk of delirium, with each additional 10 min of intraoperative hypocapnia exposure increasing the risk of delirium by 30% (OR = 1.30) (Ahrens et al., 2023). Mechanistically, hypocapnia induces cerebral vasoconstriction, leading to reduced cerebral blood flow, which may cause covert ischemia in brain tissue and interfere with neuronal energy metabolism and excitability balance.

The impact of hypercapnia on PND presents a complex dual nature. Randomized controlled trials indicate that mild controlled hypercapnia (PaCO_2_ 50–55 mmHg) can significantly increase intraoperative cerebral oxygen saturation by approximately 5–7%, demonstrating physiological benefits in improving brain oxygenation (Wong et al., 2020). However, this benefit has not been consistently validated in clinical outcomes. Recent studies have found that while controlled hypercapnia (EtCO_2_ 40–50 mmHg) can improve brain oxygen saturation, it fails to reduce the incidence of postoperative delirium (Li et al., 2025). Meanwhile, basic research has clearly revealed hypercapnia’s direct neurotoxicity. The decrease in cerebrospinal fluid pH caused by hypercapnia (Zhou et al., 2023) directly modulates the gating of voltage-gated sodium channels (Nav1.2) and TWIK-related acid-sensitive potassium channels (TASK-1/3), resulting in an overall reduction of neuronal excitability by 30–40% (O'Donohoe et al., 2019; Linden et al., 2007). Additionally, the release of presynaptic vesicles is significantly reduced (Tachibana et al., 2013), particularly affecting glutamatergic synapses, which enhance inhibitory neural impulse transmission (Sylvestre et al., 2022), exacerbating postoperative sedation and consciousness disorders. Maintaining moderate hypercapnia (PaCO_2_ ≈ 60 mmHg) for 2 h can lead to a suppression of long-term potentiation (LTP) amplitude in the mouse hippocampus by approximately 45%, and cause a decline in spatial memory performance by about 26% (Tachibana et al., 2013; Tregub et al., 2023). This indicates that hypercapnia exceeding the physiological compensation range—that is, the body’s ability to maintain acid–base balance—directly affects ion channel function and has clear neurotoxicity.

It is noteworthy that clinical observational studies have found that, compared to the clear risks of hypocapnia, mild to moderate hypercapnia during surgery (PaCO_2_ > 45 mmHg) is not significantly associated with an increased risk of delirium (Ahrens et al., 2023). Hypocapnia poses a clear risk, while hypercapnia has both physiological benefits and potential neurotoxicity, suggesting that in clinical practice, avoiding hypocapnia caused by excessive ventilation may be prioritized over concerns regarding mild hypercapnia.

#### 3.3.3 Pulmonary inflammation with nerve injury

Pulmonary infection not only leads to a systemic inflammatory response but also exacerbates cognitive function impairment by affecting oxygenation status and increasing neuroinflammation (Gao et al., 2023). During pulmonary infection, pathogen-associated molecular patterns (PAMPs) activate alveolar macrophages (Gao et al., 2023; Ryu et al., 2023). These activated macrophages release pro-inflammatory factors such as IL-1β, IL-6, and TNF-α, which can enter the CNS through disrupted barriers, directly damaging neurons and synaptic function (Yang et al., 2024; Dalile et al., 2019; Llansola et al., 2024). Additionally, pulmonary infection causes hypoxia, and the abnormal or excessive activation of HIF-1α can disrupt the integrity of the BBB, promoting inflammatory cell infiltration and further exacerbating neural damage (Arias et al., 2023). Animal experiments have shown that 24 h after infection with *Streptococcus pneumoniae*, the activation of microglia in the hippocampal region of mice is significantly increased, and levels of pro-inflammatory cytokines such as TNF-α and IL-6 also rise significantly, which is accompanied by a decline in spatial memory ability (Zhang et al., 2021). Furthermore, studies targeting human populations have confirmed that patients infected with respiratory viruses such as SARS-CoV-2 (COVID-19) and SARS-CoV exhibit significant activation of inflammatory signaling pathways in their cerebrospinal fluid (Reinhold et al., 2023; Jarius et al., 2022), along with a marked increase in the incidence of acute cognitive dysfunction (with a pooled incidence rate of delirium as high as 21.7%) (Pranata et al., 2021). This highlights the significant impact of pulmonary infection on brain function through systemic inflammation and other mechanisms.

**Figure 4:**
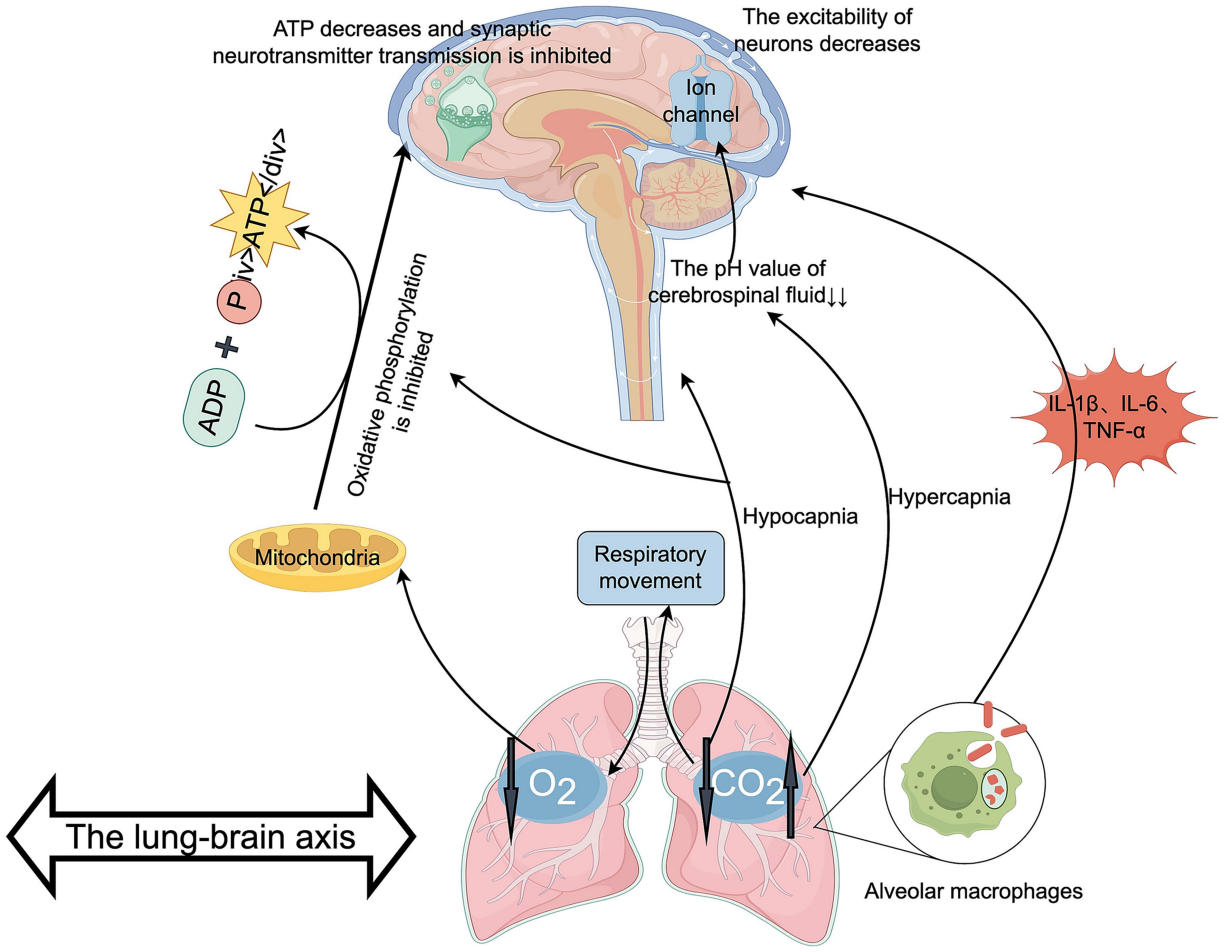
The role of the lung-brain axis in PND. Authors declare no competing interests. Graphical figures were created with FigDraw software (https://www.figdraw.com/, accessed on August 31, 2025). Copyright Code: UYTOO67d86.

These mechanisms work synergistically to form a vicious cycle. Hypoxia exacerbates the inflammatory response, which further impairs lung function. Meanwhile, hypercapnia or hypocapnia resulting from reduced lung function aggravates cerebral metabolic disorders, further promoting the occurrence of PND (Figure 4). Clinical interventions should comprehensively improve oxygenation, optimize ventilation strategies, and control inflammation to interrupt the pathological signaling along the lung-brain axis.

### 3.4 The role of the heart-brain axis in PND

There is also a significant association between cardiovascular health and cognitive function. Specifically, heart disease may affect cognitive function around the time of surgery—known as perioperative cognitive function—through various mechanisms. These include reduced cerebral blood flow (CBF), inflammation, and oxidative stress.

#### 3.4.1 Heart failure reduces cerebral blood-flow and causes brain energy deficit

In patients with cardiac insufficiency during the perioperative period, the decrease in cardiac output inevitably leads to a reduction in CBF, leading to PND (Ju et al., 2023; Bowden et al., 2022). Cardiac-origin cerebral hypoperfusion damages neurons through dual pathways. Firstly, there is the direct energy crisis, which refers to the state of cerebral hypoperfusion leading to downregulation of neuronal glucose transporter GLUT3 expression (Kisler et al., 2017), reduced activity of mitochondrial complex IV (Xie et al., 2013), and decreased ATP production (Bhushan et al., 2021), ultimately resulting in a supply–demand imbalance of brain energy. Secondly, the decoupling of the neurovascular unit (NVU) occurs (Iadecola, 2017). The reduction in CBF velocity decreases the shear stress on the vessel wall. This leads to a reduction of up to 55% in the expression of the key tight junction protein claudin-5 of the BBB through the PECAM-1/Gαq/PLCβ pathway (Ma et al., 2023). This disrupts the structural and functional connections of the NVU, thereby increasing the permeability of the BBB to peripheral toxins, which can then enter the CNS and damage neurons. Moreover, imaging studies have confirmed that under hypotensive stress, delirium-related brain regions, such as the hippocampus and cingulate gyrus, exhibit significant hypoperfusion, as evidenced by typical hypoperfusion imaging signals (Mutch et al., 2020). This directly proves that hemodynamic disturbances can lead to hypoperfusion in delirium-related brain regions (Mutch et al., 2020). Importantly, clinical intervention data show that for patients with bradycardia, improving cardiac output can enhance cognitive function scores measured by standardized neurocognitive tests by approximately 21%, accompanied by a reduction in serum IL-1β concentration of about 25% (Martis et al., 2021).

#### 3.4.2 Cardiac-cerebral chronic inflammatory cascade

The heart also affects the CNS through the “heart-brain inflammation axis.” Cardiac vagal afferent fibers express IL-1R1 and TLR4 receptors, which can transmit peripheral inflammatory signals to the solitary nucleus (Lammers-Lietz et al., 2025). This process segmentally activates microglia, resulting in a 2-3-fold increase in Iba-1 positive cells in the hippocampus (Xia et al., 2024) and significantly elevates the level of CNS inflammation. Patients who experience cardiac arrest (CA) and achieve the return of spontaneous circulation (ROSC) often suffer from severe neuroinflammation due to global cerebral ischemia–reperfusion injury, with excessive activation of microglia in multiple brain regions (Chen et al., 2025). Furthermore, patients with heart failure generally exhibit a systemic inflammatory state (serum IL-6 is commonly elevated) (Gui and Rabkin, 2023; Tromp et al., 2018), and surgical trauma can further amplify this inflammatory response. Baseline data from a multicenter randomized controlled trial indicate that in the control group, patients who did not receive neuroprotective interventions showed that the serum level of the neuroinflammatory marker S100β can increase to approximately 2.8 times the preoperative baseline value within 24 h post-surgery (Liu et al., 2023); the magnitude of this increase is significantly correlated with the incidence of postoperative delirium (*p* < 0.01) (Dunne et al., 2021). Long-term patients with cardiac insufficiency experience a 1–2% annual reduction in hippocampal volume, with a faster decline in memory scores compared to peers, which may also be related to chronic inflammation (Traub et al., 2024).

**Figure 5:**
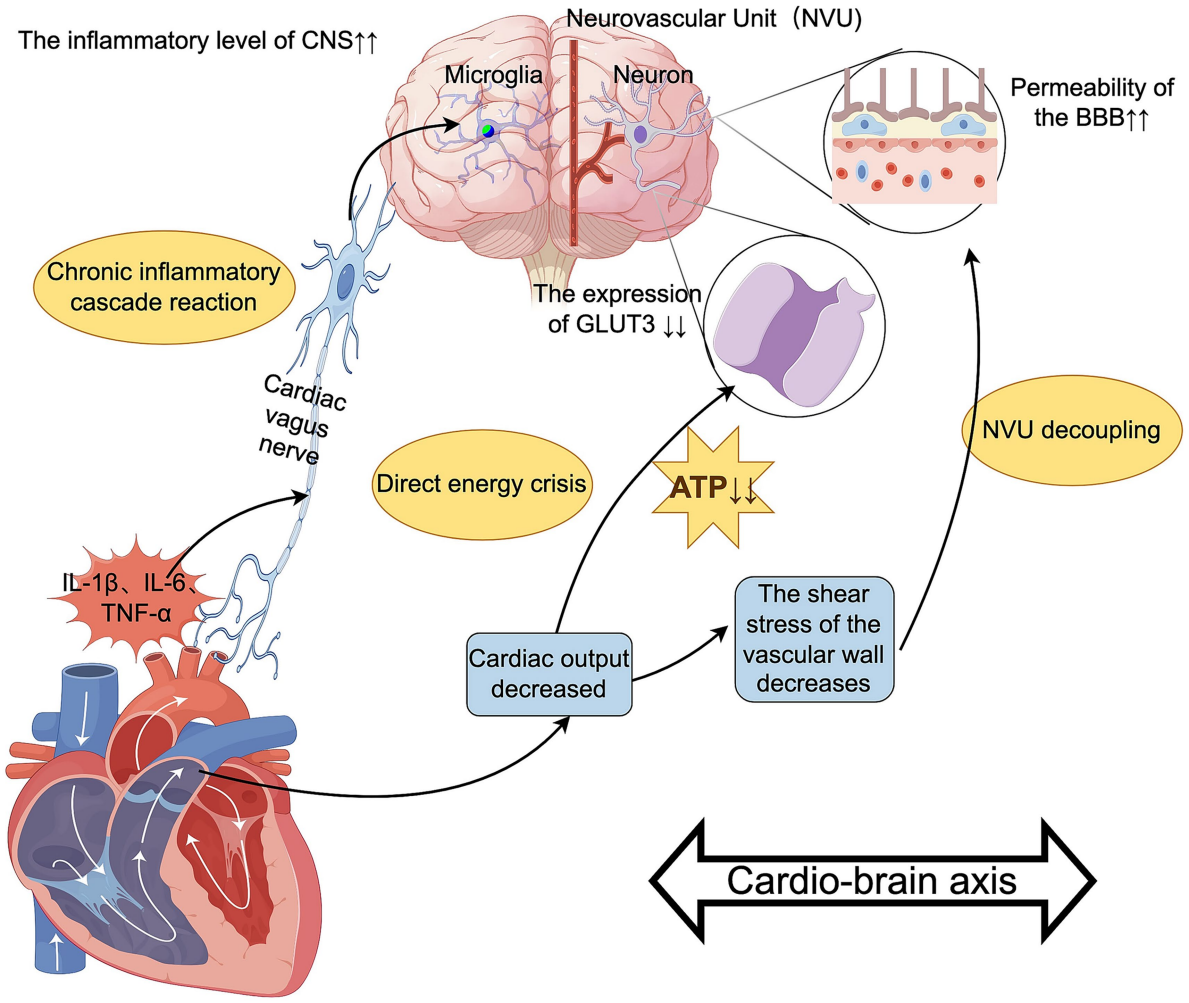
The role of the heart-brain axis in PND. Authors declare no competing interests. Graphical figures were created with FigDraw software (https://www.figdraw.com/, accessed on August 31, 2025). Copyright Code: SUYYT8dfd7.

Insufficient cerebral blood flow leads to neuronal energy metabolism disorders, and chronic inflammation activates microglia through cytokines, causing CNS inflammation. These two factors intertwine, forming a vicious cycle: low perfusion exacerbates inflammation and oxidative stress, which in turn further impairs vascular regulatory function, disrupts energy metabolism, and ultimately plays an important role in the occurrence and development of PND in the heart-brain axis (Figure 5).

### 3.5 The role of the spleen-brain axis in PND

The spleen, as an important immune regulatory organ, may significantly impact the occurrence and development of PND. It does so by regulating the systemic inflammatory response and immune cell migration. Supporting this, studies have found that mice with impaired spleen function, or those that underwent splenectomy, have an increased susceptibility to PND, suggesting that the spleen plays a crucial role in maintaining perioperative neural homeostasis (Liu Y. et al., 2024).

#### 3.5.1 Spleen-mediated modulation of CNS inflammation

The mechanism by which the spleen-brain axis regulates PND involves multiple pathways, the most important of which may be mediating the balance between peripheral inflammation and the CNS. Because the spleen is the largest lymphoid organ in the body, perioperative stress causes it to release inflammatory mediators that promote neuroinflammation, leading to cognitive decline (Feng et al., 2017). The spleen can influence the progression of neuroinflammation by regulating the polarization of peripheral immune cells into M1 and M2 macrophage phenotypes (Aiello et al., 2020); M1 macrophages promote pro-inflammatory responses, while M2 macrophages have anti-inflammatory and neuroprotective effects. The dual role of the splenic branch of the vagus nerve in anti-inflammatory and pro-inflammatory responses also plays an important role. On one hand, the spleen exerts neuroprotective effects through the cholinergic anti-inflammatory pathway via the vagus nerve. The efferent branches of the splenic vagus nerve release acetylcholine, which binds to α7nAChR on splenic macrophages, inhibiting NF-κB activation and reducing the release of pro-inflammatory factors from the spleen (Pavlov et al., 2018). Animal studies have shown that electrical stimulation of the vagus nerve to activate the cholinergic anti-inflammatory pathway in the spleen can significantly reduce TNF-α levels in the spleen and brain (by approximately 40%) (Mihaylova et al., 2012), and correspondingly improve postoperative cognitive function, with behavioral test scores increasing by about 35% (Mihaylova et al., 2012). Meta-analysis also confirmed that animal behavioral test performances showed highly significant improvement compared to the control group, with a large overall effect size (SMD = −2.87, *p* < 0.001) (Zhang H. et al., 2022). On the other hand, peripheral inflammatory mediators activate specific receptors on the afferent fibers of the splenic vagus nerve, transmitting peripheral inflammatory signals to brain regions associated with cognition such as the hippocampus and prefrontal cortex via the nucleus of the solitary tract. This leads to exacerbation of local neuroinflammation and thereby impairs synaptic plasticity (Ben-David et al., 2020). In animal experiments, aged rats significantly exhibited a postoperative decline in spatial learning and memory ability of about 30–40% and impaired executive function (Zhang et al., 2023b). The splenic vagus nerve pathway is a complex bidirectional signaling pathway. Its efferent branches exert anti-inflammatory and neuroprotective effects, while its afferent branches are responsible for transmitting pro-inflammatory signals, serving as a key bridge mediating peripheral inflammation leading to CNS inflammation and cognitive impairment (Zhang et al., 2023b). In summary, the spleen is a critical link in the amplification of neuroinflammatory responses.

#### 3.5.2 Exosomes from the spleen remotely regulate brain function

The spleen, as a complex immune organ, contains various immune cells, such as splenic macrophages, dendritic cells, mesenchymal stem cells (MSCs) and B lymphocytes. These different types of cells can remotely regulate gene expression in the brain by secreting exosomes carrying microRNAs (miRNAs) and other bioactive molecules. These exosomes affect synaptic plasticity and neuronal survival (Pitt et al., 2016; Muntasell et al., 2007), thereby playing a dual regulatory role in both the initiation and progression of PND. Under physiological conditions, the spleen can secrete exosomes with neuroprotective effects: macrophage-derived miR-146a-5p can downregulate TLR4 expression in microglia, alleviating neuroinflammation (Zhang et al., 2023a); MSC-derived miR-125b can counteract Aβ-induced synaptic damage and promote synaptic plasticity (Zhang J. et al., 2022). Moreover, Deng et al. (2024) and others found that exosomes derived from splenic MSCs have a positive effect on nerve regeneration and cognitive function recovery. However, under pathological conditions, surgical trauma can activate splenic immune cells (especially red pulp macrophages and marginal zone B cells), leading to a large release of pro-inflammatory exosomes. These exosomes carry pro-inflammatory miRNAs such as miR-155-5p and miR-193a-5p (Fang et al., 2024). They can be taken up by neurons and microglia, where they inhibit the expression of BDNF. This inhibition causes sustained amplification of neuroinflammation and mediates a decline in cognitive function levels. Clinical studies have found that the level of exosomal miR-193a-5p in the plasma of PND patients after surgery is several times higher (approximately 3–5 times) than that of non-PND patients, and its expression level is positively correlated with the duration of delirium (*R* = 0.51) (Xu et al., 2025), suggesting it could serve as a potential biomarker. This indicates that perioperative stress leads to a shift in the “pro-inflammatory/anti-inflammatory” balance of splenic exosomes toward a pathological direction, increasing central inflammation levels and promoting the occurrence of PND.

**Figure 6:**
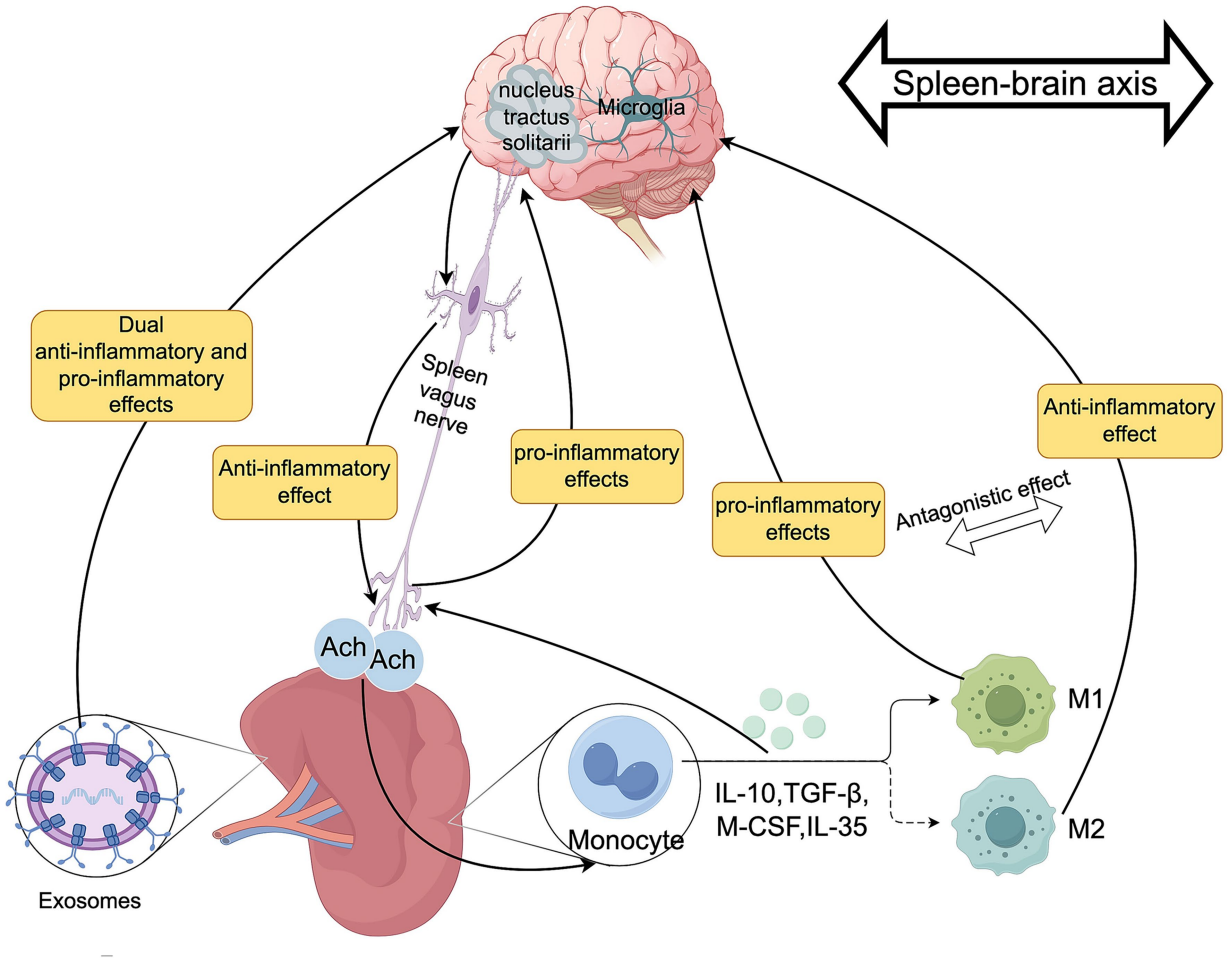
The role of the spleen-brain axis in PND. Authors declare no competing interests. Graphical figures were created with FigDraw software (https://www.figdraw.com/, accessed on August 31, 2025). Copyright Code: USPTR77e10.

In summary, the spleen-brain axis participates in the occurrence and development of PND through various mechanisms, including regulating inflammatory balance, neuroendocrine functions, and extracellular vesicles (Figure 6).

### 3.6 The role of the kidney-brain axis in PND

The kidney, as an important metabolic and endocrine organ, is significantly associated with cognitive impairment when they become dysfunctional. Studies have shown that the incidence of PND is significantly increased in patients with chronic kidney disease (CKD) (Li et al., 2024). Additionally, the risk of dementia in patients with CKD is 2 to 3 times higher than in the general population (Bugnicourt et al., 2013). These findings suggest that chronic kidney disease may promote cognitive decline through various pathways, with the main mechanisms possibly related to the accumulation of uremic toxins, inflammatory responses, oxidative stress, endothelial dysfunction, and neuroendocrine disorders.

#### 3.6.1 Neurotoxicity induced by uremic toxins

Various uremic toxins accumulated during renal function decline (Li et al., 2021; Yamamoto, 2019), including small, water-soluble molecules, middle molecular toxins, and protein-bound toxins, can damage the integrity of the BBB and directly injure neurons, ultimately promoting the progression of PND. The protein-bound toxin p-cresol sulfate (PCS) activates microglia by promoting the release of TNF-α and IL-1β, inducing neuroinflammation (Azargoonjahromi et al., 2025). In contrast, indole-3-propionic acid can inhibit the expression of synaptic plasticity-related proteins in the hippocampus, such as postsynaptic density protein 95 (PSD-95) and BDNF, impairing learning and memory function (Sun et al., 2021). β2-microglobulin (β2-MG) (Yamamoto, 2019), a middle molecular toxin, leaks into the CNS through the BBB, promoting the hyperphosphorylation of tau protein and facilitating the deposition of amyloid proteins in the brain alongside amyloid-beta (Aβ). Studies have found that blood levels of β2-MG in patients with renal insufficiency are negatively correlated with MMSE cognitive scores (β ≈ −0.10, *p* < 0.001) (Ke et al., 2025). In addition, the decline in renal clearance function leads to the accumulation of small, water-soluble molecules such as homocysteine (Hcy), which exacerbates oxidative stress by activating the NMDAR / ROS pathway, increasing the apoptosis rate of neurons by approximately 20–40% (Luzzi et al., 2022).

#### 3.6.2 Dysfunction of the kidney-brain axis mediated by neuroendocrine disorders

The endocrine disorders caused by renal insufficiency represent another important mechanism of the kidney-brain axis. Erythropoietin (EPO), secreted by the kidneys, has neuroprotective effects; however, its level decreases during renal insufficiency, which can lead to reduced neurogenesis in the hippocampus (Vittori et al., 2021). A phase III clinical trial showed that treatment with recombinant human EPO (rhEPO) in patients with chronic kidney disease increased their MoCA scores by an average of about 2.5 points (Ehrenreich et al., 2009). Additionally, secondary hyperparathyroidism (SHPT) is a significant complication of renal insufficiency. The hyperphosphatemia and elevated fibroblast growth factor 23 (FGF23) caused by SHPT may induce brain edema by disrupting cerebral vascular autoregulation and may also inhibit neuronal energy metabolism (Gong et al., 2020; Drew et al., 2020). Clinical studies have confirmed that preoperative vitamin D deficiency is an independent risk factor for PND in elderly patients, increasing the risk by 60 to 150% (*OR* = 1.6–2.5) (Hung et al., 2022). This association likely results from insufficient activity of renal 1α-hydroxylase, which leads to low levels of active vitamin D in the brain and subsequently reduces the synthesis of neurotrophic factors (Hung et al., 2022).

#### 3.6.3 Kidney-derived exosomes mediate neuronal injury

In recent years, exosome-mediated inter-organ communication has received widespread attention. Under stress conditions such as surgical trauma and ischemia–reperfusion injury, the kidneys can release a large number of exosomes (Lv et al., 2020) into the circulatory system. These exosomes carry specific miRNAs, such as miR-21-5p and miR-155, and express specific integrins on their surface, such as ITGα6β4 and ITGαvβ5. This enables the exosomes to selectively target brain endothelial cells and cross the BBB through transcytosis. Once in the brain, they regulate multiple inflammatory pathways and interfere with neuronal energy metabolism, which plays an important role in cognition and memory functions (Zang et al., 2019; Ianni et al., 2024). For example, pro-inflammatory miRNAs such as miR-155 carried by renal exosomes are key mediators in regulating neuroinflammation. Specifically, miR-155 (Zingale et al., 2021) targets the zinc finger protein Tristetraprolin (TTP), thereby enhancing the stability of IL-6 and IL-8 mRNA and further amplifying the neuroinflammatory cascade. In addition to pro-inflammatory effects, renal exosomes can also interfere with neuronal energy metabolism through molecules such as miR-668 (He and Zhang, 2020). In AKI animal models, exosomal miR-668 reduces the membrane localization of glucose transporters GLUT1 and GLUT3 by inhibiting the expression of insulin-like growth factor 1 receptor (IGF1R) in the hippocampus, leading to insufficient energy supply for neurons and impaired synaptic plasticity (Li S. et al., 2022). This metabolic disorder can further exacerbate Aβ deposition and tau protein hyperphosphorylation (Lang et al., 2023), forming pathological features similar to Alzheimer’s disease. It is noteworthy that renal exosomes have bidirectional regulatory potential. Under physiological conditions, the kidneys secrete exosomes carrying anti-inflammatory miRNAs such as miR-146a-5p (Zhang et al., 2023a; Valiuliene et al., 2023), which alleviate neuroinflammation by inhibiting the IRAK1/TRAF6 signaling pathway. However, during perioperative stress conditions, this protective mechanism is often disrupted, resulting in a predominance of pro-inflammatory exosomes. Although there is currently insufficient clinical research directly targeting the association between renal-derived exosomes and PND, a large body of basic research evidence suggests that renal exosomes are a promising mediator connecting peripheral organ injury and CNS function, making them an important focus for future exploration as early warning biomarkers or intervention targets for PND.

In addition, the kidneys also regulate the brain’s neuroinflammatory response through the renin-angiotensin system (RAS) (Chagas et al., 2025). Angiotensin II (Ang II) excessively activates the AT1 receptor (AT1R), promoting NADPH oxidase-dependent oxidative stress in the nervous system. Angiotensin receptor blockers (ARBs), such as losartan, can alleviate postoperative cognitive impairment (Althammer et al., 2023). These findings indicate that renal inflammatory responses are also key contributors to the development of PND.

**Figure 7:**
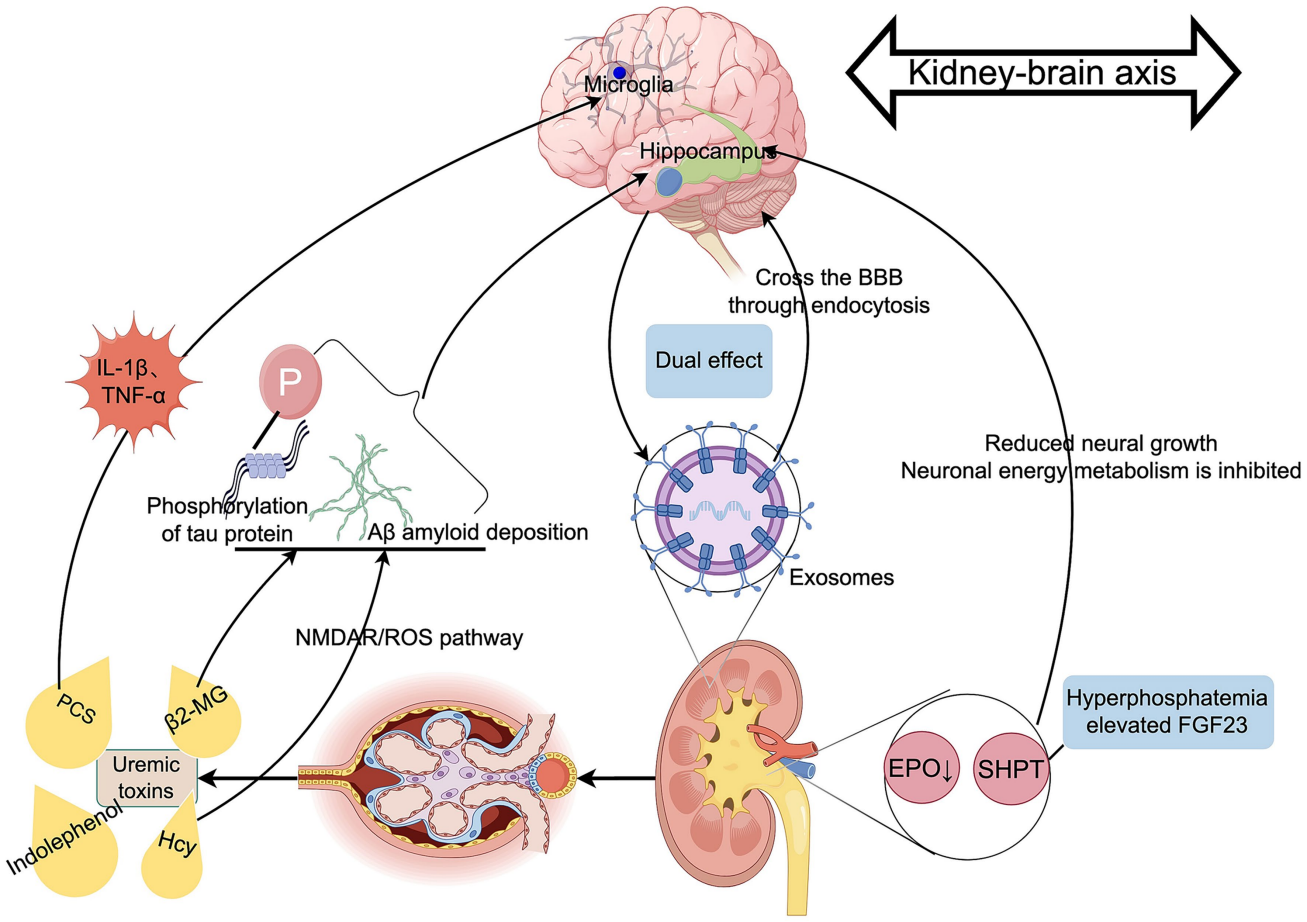
The role of the kidney-brain axis in PND. Authors declare no competing interests. Graphical figures were created with FigDraw software (https://www.figdraw.com/, accessed on August 31, 2025). Copyright Code: OPPWY5c155.

In summary, the kidney–brain axis participates in the pathological process of PND through multiple mechanisms, including barriers involved in toxin clearance, endocrine disorders, and exosome signaling (Figure 7). The therapeutic potential of renal protection strategies in the prevention and treatment of PND may be a focus of future research.

## 4 The relationship between MODS and PND

**Figure 8:**
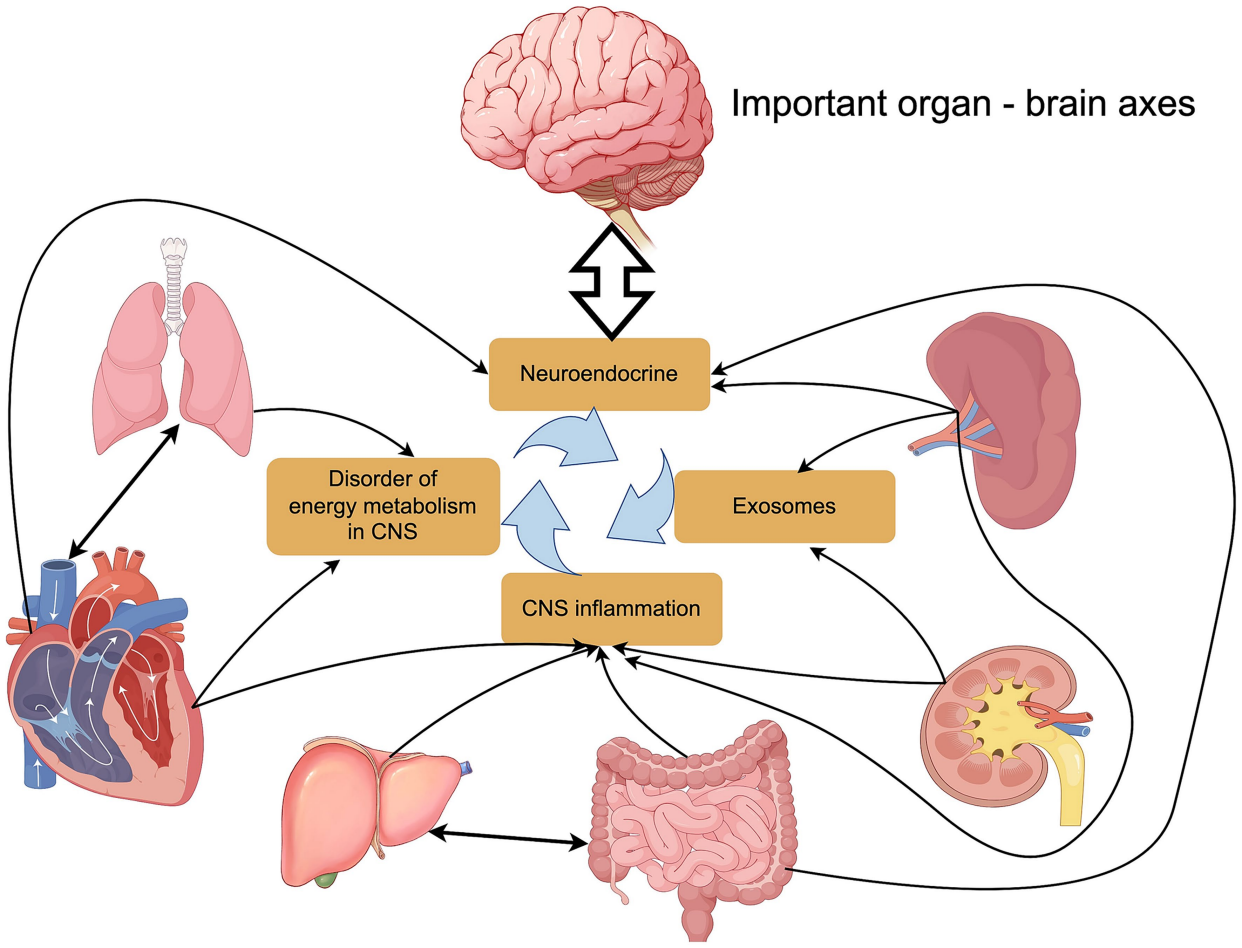
The relationship between MODS and PND. Authors declare no competing interests. Graphical figures were created with FigDraw software (https://www.figdraw.com/, accessed on August 31, 2025). Copyright Code: IWIORd8e99.

The above text elaborates on the relationship between the gut-brain axis, liver-brain axis, lung-brain axis, heart-brain axis, spleen-brain axis, kidney-brain axis, and PND. We can say that the occurrence of PND is essentially the result of an imbalance in the multi-organ functional network, rather than a direct result of a single organ dysfunction. Recent studies have revealed that the functional axes of various organs (gut, liver, lung, heart, spleen, and kidney) work together through the synergistic effect of “inflammation-metabolism-microcirculation,” forming the pathophysiological basis of PND. The systemic stress response triggered by surgical trauma can lead to distant organ damage, consequently creating a complex pathological positive feedback loop that promotes the occurrence and progression of PND. Clinical data show that critically ill patients with multiple organ dysfunction have higher levels of neuroinflammation and poorer long-term cognitive function compared to ordinary patients (Hughes et al., 2018) (Figure 8).

From the above discussion on the influence of each axis on PND, it can be seen that the cascading amplification of the inflammatory network is the core mechanism promoting the occurrence and development of PND. The LPS released from the damaged intestinal epithelial barrier can activate hepatic Kupffer cells to produce IL-6 (Zhang X. et al., 2022). This IL-6 then amplifies the inflammatory signal by activating pulmonary vascular endothelial cells. Meanwhile, pro-inflammatory exosomes, such as miR-155, released from the spleen can penetrate the blood–brain barrier, whose permeability is increased due to inflammation (Ke et al., 2023). The decline in renal excretory function leads to the accumulation of uremic toxins, such as indoxyl sulfate, which inhibits microglial anti-inflammatory polarization (Yamamoto, 2019). This synergistic effect of the “gut-liver-lung-spleen-kidney” inflammatory axis results in significantly higher activation levels of microglia in the hippocampus of patients with multiple organ dysfunction compared to those with single organ injury (Castro et al., 2022). Meanwhile, heart failure exacerbates the neuronal energy deficit induced by inflammation by reducing CBF perfusion (Jinawong et al., 2021).

The cross-impact of metabolic disorders also plays a key role. Liver ammonia metabolism disorders and renal acidosis synergistically alter the pH in the brain, inhibiting the activity of glutamate decarboxylase and leading to suppression of GABAergic neuronal function (Hadjihambi et al., 2017; Luzzi et al., 2022). Hypoxemia caused by pulmonary gas exchange disorders can reduce ATP production in the hippocampus by 57% (Ding et al., 2018; Zhang K. et al., 2024; Jing et al., 2022), while the reduction of SCFAs due to gut microbiota dysbiosis further exacerbates mitochondrial dysfunction (Su et al., 2023). A comprehensive metabolomics analysis of the hippocampus in mice with multiple organ dysfunction syndrome (MODS) indicates that the levels of key intermediates in the tricarboxylic acid cycle (such as α-ketoglutarate) are significantly lower than those in the control group. This metabolite level change is accompanied by an increase in L-lactate levels (Xu et al., 2022), suggesting that the energy metabolism pathways in the brain of MODS patients are extensively disrupted. Furthermore, the chronic cerebral hypoperfusion state caused by reduced cardiac output can exacerbate neuronal energy supply deficiency by downregulating the expression of the glucose transporter GLUT3 (Kisler et al., 2017; Bhushan et al., 2021).

Exosome-mediated communication between organs and the brain has emerged as a new area of great interest in recent years. After injury, various organs release organ-specific exosomes, forming a cascade of pathological signal transmission that triggers multiple downstream effects: cardiac-derived miR-208a increases blood–brain barrier permeability by inhibiting the expression of claudin-5, “opening the door” for subsequent inflammatory attacks (Han et al., 2023); liver exosomal miR-122-5p promotes the polarization of microglia toward a pro-inflammatory phenotype (M1 type) (Yu et al., 2023), creating an “inflammatory storm” within the brain; additionally, renal miR-21-5p (Zang et al., 2019) inhibits neuronal synaptic plasticity, directly “cutting off” the communication lines of neurons. This multi-targeted, synergistic attack ultimately leads to the collapse of neural function (Kalani et al., 2014).

Based on the understanding of the multi-organ functional network, perioperative multi-organ protection strategies should adopt an integrated intervention approach to prevent the occurrence of PND. These strategies include inflammation regulation: modulating gut microbiota and early postoperative use of low-dose corticosteroids, among others (Tang et al., 2024; Li et al., 2023); metabolic support: effectively enhancing oxygen delivery and preoperative supplementation of ketone bodies, among others (Wang et al., 2022; Titlestad et al., 2024); microcirculation protection: optimizing fluid management and remote ischemic preconditioning, among others (Pearse et al., 2014; Sridharan et al., 2019); and neuro-specific interventions: using dexmedetomidine perioperatively and intranasal insulin administration, among others (Li et al., 2020; Yang et al., 2025). Multiple studies have confirmed that the integrated strategy of multi-organ system support combined with bundled treatment protocols can significantly reduce the incidence of PND in high-risk patients and notably shorten the average length of hospital stay (Ahrens et al., 2023; Hou et al., 2025). Clinical interventions need to adopt a multi-target integrated strategy, and future research should focus on the temporal patterns of organ interactions and develop PND prediction models based on multi-organ function scoring.

## 5 Conclusion and future work

The occurrence of PND is a complex, multifactorial process involving interactions among multiple critical organs, including the brain. Through in-depth studies of axes including the gut-brain axis, liver-brain axis, lung-brain axis, heart-brain axis, spleen-brain axis, and kidney-brain axis, we can gain a more comprehensive understanding of the pathophysiological mechanisms of PND. By investigating these mechanisms, researchers not only identify critical biomarkers for recognizing high-risk patients but also lay the foundation for the development of clinical intervention strategies.

When analyzing the viewpoints and findings of different studies, we observed distinct differences and complexities in the interactions between various organs and the brain. For example, changes in gut microbiota may affect brain function through neuroimmune pathways, and metabolic alterations in the liver may exacerbate cognitive impairment through inflammatory responses. Therefore, when formulating patient management strategies, clinicians need to comprehensively consider factors such as gut microbiota, liver metabolism, and neuroimmune interactions to achieve more effective interventions.

Future research on the organ-brain axis and PND should move beyond the isolated description of a single axis and shift toward constructing a dynamic, interactive “multi-organ-brain network” paradigm. At the same time, future studies may focus on the following cutting-edge directions: (1) Analyzing the temporal and hierarchical relationships between organs by clarifying whether there is a sequence of activation among different organ-brain axes during the perioperative period—such as whether intestinal barrier dysfunction initiates inflammation—or a core-periphery hierarchical structure. Additionally, utilizing multi-omics technologies (proteomics, metabolomics, microbiome analysis) to longitudinally track patient samples and map the “event map” of the pathophysiological process of PND. (2) Defining the overlooked key scientific issue—the pivotal role of the NVU; the NVU is the common pathway and integration platform for all organ-brain axis signals entering the brain. Future research needs to explore how harmful signals from different organs (such as inflammatory factors, uremic toxins, exosomes) synergistically disrupt NVU function, leading to changes in blood–brain barrier permeability and an imbalance in brain homeostasis, which is currently a weak point in research. (3) Developing precise prediction and intervention strategies based on multi-organ risk factors by integrating clinical data and biological markers (such as organ-specific exosomes, inflammatory factor profiles) to construct a multi-organ functional risk scoring system for PND. Based on this, exploring the value of targeted interventions (such as specific probiotics, vagus nerve stimulation, exosome therapy) on key pathways like the “gut-liver-brain axis” and “spleen-brain axis” in the precise prevention of PND.

Through the above research, we hope to integrate the currently fragmented concept of “axis” into a systematic “network medicine” model. This integration will provide a novel theoretical framework for achieving precise early warning and individualized prevention and treatment of PND, thereby enabling better management of patients’ perioperative health.

## 6 Limitations

This review has some limitations. First, the literature search mainly focused on the PubMed and CNKI databases, and the search terms were primarily in Chinese and English. This approach may have led to the omission of important studies published in other languages, such as Japanese and German, or in other regional databases. Such omissions could introduce potential selection bias. Second, given that research on some organ-brain axes, such as the spleen-brain axis and kidney-brain axis, is still in its early stages, existing evidence mainly comes from animal experiments or small-sample clinical observational studies, lacking support from large-scale, multi-center prospective research data. Therefore, caution should be exercised when demonstrating causal associations and mechanistic insights. In addition, although the review mentions interactions between various axes, it fails to quantitatively assess the specific contributions of these interactions among multiple axes—whether synergistic or antagonistic—to the risk of PND, and its integrative pathophysiological model still requires improvement. Another obvious limitation is the significant heterogeneity in the diagnosis and evaluation of PND across different studies. This heterogeneity includes inconsistencies in the neuropsychological scales used (MMSE, MoCA), follow-up time points, and the application of diagnostic criteria such as DSM-5. These differences may severely affect the comparability of results and the generalizability of conclusions. Finally, the multi-organ protection strategies proposed based on existing evidence, such as regulating microbiota and targeting exosomes, mostly stem from preclinical studies. Their efficacy and safety in clinical translation remain to be validated by future high-quality randomized controlled trials (RCTs).
